# Big Data Health Care Platform With Multisource Heterogeneous Data Integration and Massive High-Dimensional Data Governance for Large Hospitals: Design, Development, and Application

**DOI:** 10.2196/36481

**Published:** 2022-04-13

**Authors:** Miye Wang, Sheyu Li, Tao Zheng, Nan Li, Qingke Shi, Xuejun Zhuo, Renxin Ding, Yong Huang

**Affiliations:** 1 Engineering Research Center of Medical Information Technology West China Hospital of Sichuan University Ministry of Education Chengdu, Sichuan Province China; 2 Department of Endocrinology and Metabolism MAGIC China Centre, Cochrane China Centre West China Hospital, Sichuan University Chengdu China

**Keywords:** big data platform in health care, multisource, heterogeneous, data integration, data governance, data application, data security, data quality control, big data, data science, medical informatics, health care

## Abstract

**Background:**

With the advent of data-intensive science, a full integration of big data science and health care will bring a cross-field revolution to the medical community in China. The concept *big data* represents not only a technology but also a resource and a method. Big data are regarded as an important strategic resource both at the national level and at the medical institutional level, thus great importance has been attached to the construction of a big data platform for health care.

**Objective:**

We aimed to develop and implement a big data platform for a large hospital, to overcome difficulties in integrating, calculating, storing, and governing multisource heterogeneous data in a standardized way, as well as to ensure health care data security.

**Methods:**

The project to build a big data platform at West China Hospital of Sichuan University was launched in 2017. The West China Hospital of Sichuan University big data platform has extracted, integrated, and governed data from different departments and sections of the hospital since January 2008. A master–slave mode was implemented to realize the real-time integration of multisource heterogeneous massive data, and an environment that separates heterogeneous characteristic data storage and calculation processes was built. A business-based metadata model was improved for data quality control, and a standardized health care data governance system and scientific closed-loop data security ecology were established.

**Results:**

After 3 years of design, development, and testing, the West China Hospital of Sichuan University big data platform was formally brought online in November 2020. It has formed a massive multidimensional data resource database, with more than 12.49 million patients, 75.67 million visits, and 8475 data variables. Along with hospital operations data, newly generated data are entered into the platform in real time. Since its launch, the platform has supported more than 20 major projects and provided data service, storage, and computing power support to many scientific teams, facilitating a shift in the data support model—from conventional manual extraction to self-service retrieval (which has reached 8561 retrievals per month).

**Conclusions:**

The platform can combine operation systems data from all departments and sections in a hospital to form a massive high-dimensional high-quality health care database that allows electronic medical records to be used effectively and taps into the value of data to fully support clinical services, scientific research, and operations management. The West China Hospital of Sichuan University big data platform can successfully generate multisource heterogeneous data storage and computing power. By effectively governing massive multidimensional data gathered from multiple sources, the West China Hospital of Sichuan University big data platform provides highly available data assets and thus has a high application value in the health care field. The West China Hospital of Sichuan University big data platform facilitates simpler and more efficient utilization of electronic medical record data for real-world research.

## Introduction

### Background

Emerging technologies, such as big data, the Internet of Things, cloud computing, and artificial intelligence, are profoundly changing medical and health care service models. Health and medical data sets, which are typically large with rapid growth, diverse data structure, multidimensional value density, high requirements of data credibility, and high concerns over data security, are often called health care big data. The term *big data* is used to represent not only a technology but also a resource and a method—a big data platform is a system and a tool that integrates data, tools, apps, and service.

Driven by national policies, high-income countries in Europe, such as the United Kingdom [1], and in North America, such as the United States [2], took the lead in building big data platforms for health care [3]. For example, Britain’s comprehensive platform integrates and applies data from 12 categories, including health, medical care, transportation, and environment, to support government decision-making [3]. The Big Data Analytics Platform built by the Czech Republic meets the data analysis requirements of its national public health service [4]. In recent years, China has built a national-level health information platform that can be connected to provincial-level health information platforms for data integration [5].

In addition to national-level big data platforms in health care that are driven by policies, large health care institutions have also built big data platforms to suit their own management needs. At present, a medium-sized health care institution in China generates 1 to 20 TB of health care data, and a large health care institution generates 300 TB to 1 PB of health care data every year. Some hospitals have used big data technology to build hospital-level scientific research platforms—for example, Ninewells Hospital and Medical School in the United Kingdom developed a research data management platform [6], Asan Medical Center in South Korea developed a clinical trial management system based on integrated data [7], and the People's Hospital of Peking University in China developed a hospital-wide big data platform for clinical research [8]—or to build hospital-wide data integration platform—for example, the Third Hospital of Peking University [9] and the Second Affiliated Hospital of Guangzhou University of Traditional Chinese Medicine [10]. Most hospitals use big data technology in analytics platforms for diseases (eg, for nasopharyngeal carcinoma [11]), gastrointestinal conditions [12], cancer [13], and cardiomyopathies [14].

Meta-analyses [15-18] have found that big data platforms in health care have had great impacts on have had great impacts on medical technology, medical service quality, and medical costs, but the actual construction process is not easy, with challenges arising in data structurization, security, standardization, storage, processing, and management [15,16]. As a result, the value mined from this type of data is currently limited [17], and in China, health care data utilization is still in the early stage [18], which necessitates the improvement of health care big data governance.

### Objectives

Health care data generated in large hospitals include (1) electronic medical records of clinical diagnosis and treatment, which contain diagnoses, prescriptions, surgical treatments, and examination findings; (2) data derived from health management or clinical research activities, including follow-up information, gene sequencing data, and physical examination data; (3) data related to hospital management, including patient wait times, bed turnover rate, medical equipment utilization efficiency, and revenue; and (4) data derived from web-based diagnosis and treatment services [19]. To make better use of medical data, the first task is to build a big data platform for data collection and governance to generate high-quality data assets. This will enable in-depth data analysis and mining, as well as the formation of rules of knowledge, allowing big data methods to benefit clinical practice, scientific research, and hospital management.

In addition to the technical considerations for platform construction, data management in large hospitals must take into account by data service management. Special attention should be paid to patient privacy protection and ethical concerns on data use. A hospital data platform is generally built for a specific purpose, such as scientific research, operations, data integration, or analysis, but large hospitals often require a comprehensive data platform—one that meets needs for medical treatment, education, scientific research, and management.

The overall objective of this study was to develop and implement a health care big data platform for a large hospital in China, with data governance as the core concept, to solve difficulties related to integration, calculation, storage, standardization, and security for multisource heterogeneous medical data. This platform will integrate the data from all operation systems in a hospital to generate high-quality data assets and form a massive high-dimensional medical database that can comprehensively support the clinical activities, scientific research, and management of the hospital.

From a technical aspect, construction of a big data platform needs to solve the following problems that are unique to the health care industry: (1) integration of multisource heterogeneous data from multiple independent information systems within the hospital; (2) computing power requirements brought by the development of machine learning and deep learning during data application; (3) difficulties in analyzing and utilizing information data (due to nonuniform data standards, inconsistent use of a master patient index, and the fact that most electronic medical records in China are written in natural language, it is impossible to directly analyze and use existing information data, and as a result, semantic interoperability must be improved with the use of medical terminology) and (4) data security and patient privacy protection.

## Methods

### Overview

The West China Hospital of Sichuan University (WCH) built a health care big data platform, which is referred to as the WCH-BDP or *the platform* hereafter. WCH is a world-renowned large hospital with more than 4800 beds and approximately 15,000 outpatients on average per day. WCH’s electronic medical record system was built in 2007; therefore, it has been in use for 14 years. The hospital has more than 100 departments and sections, and their clinical activities generate a massive amount of data.

Traditional information technology can no longer handle massive amounts of data that are continuously growing, causing difficulties in effectively integrating multisource heterogeneous data, the serious problem of isolated data islands, bottlenecks in data storage and calculation, difficulties in structurally utilizing medical records written in Chinese semantics, and high technical barriers in mining data from images, videos, and files. In 2017, WCH launched a hospital-wide health care big data platform project to address these difficulties.

The project focuses on the design and development of the platform architecture and does not involve any study of clinical data, so ethical declarations are not applicable.

### Project Organization

The first step of platform construction was organizing a project management team. To ensure the performance of the platform, WCH set up 2 working groups—one for platform construction, and the other for platform management. The construction working group included the chief platform architect, information technology experts, system engineers, and data engineers. The hospital information center was responsible for building the platform. The construction working group focused on the following: (1) the application objectives of the platform, (2) data integration method and scope, (3) master patient index strategy, (4) reference standards for medical terms, (5) scope of the master data, (6) data model structure, and (7) system implementation and training. Project meetings were held weekly and status meetings were held monthly to summarize the progress of the phases until the platform was launched. The discussions in these meetings provided a solid foundation for platform design and development. After the launch of the platform, the construction working group also conducted regular training on system functions, maintenance, and the help manual.

The management working group included all stakeholders, such as the chief information officer, hospital management users, clinical users, and scientific research users. The management group was the data governance committee responsible for organizing and overseeing all data-related work, including data definitions, data benchmarking, data quality control, and data security, after the launch of the platform. The committee was in charge of formulating related management systems, work collaboration mechanisms, and procedure standardization.

### WCH-BDP Framework Design Strategy

**Figure 1:**
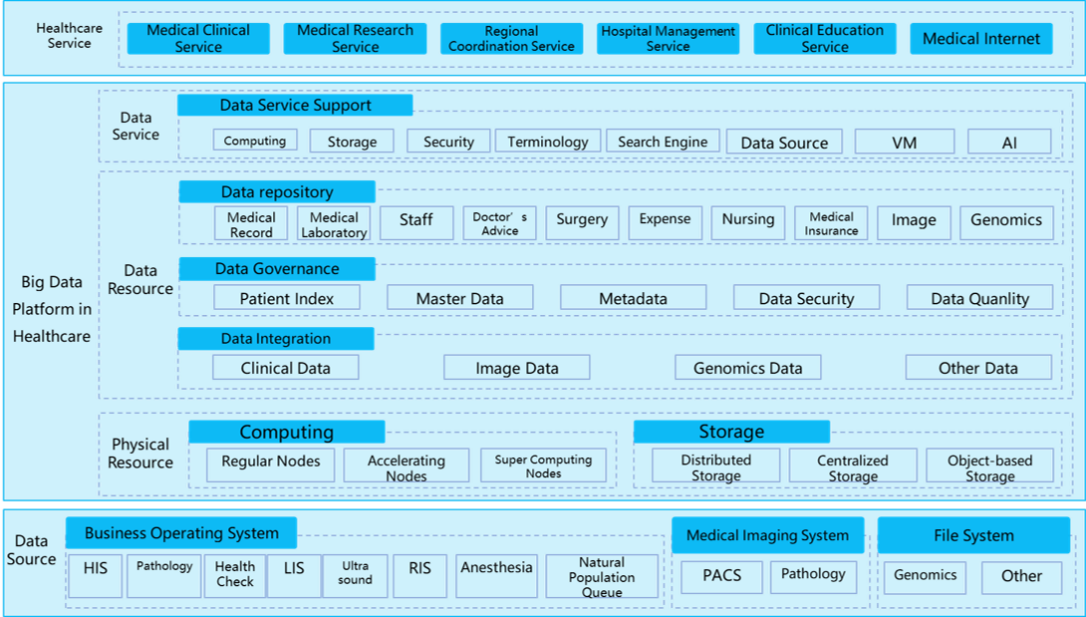
Big data platform architecture.

The convergence of multisource heterogeneous data from all operation systems in the hospital was necessary to ultimately provide data to different medical services (Figure 1). Therefore, the design strategy was to use computing and storage devices with enough capacity to integrate data into the corresponding physical resources, with separate storage and computing processes based on the characteristics of the modality (eg, clinical data, image data, or genomic data). A data repository was built using data governance methods to meet the needs of different subject fields. The platform had to be able to combine all data service supports, such as data security service, terminology service for data governance, search engine service, virtualization service, and artificial intelligence service—all of which rely on the computing power and storage capacity of physical devices and data resources. Solutions for data integration and governance were core aspects of the construction process. Data governance, which is the core of data management and the basis for standardizing disordered data into highly available data, includes master index data governance, master data governance, metadata governance, data security management, and data quality control management [10,20,21].

### Data Storage and Calculation

**Figure 2:**
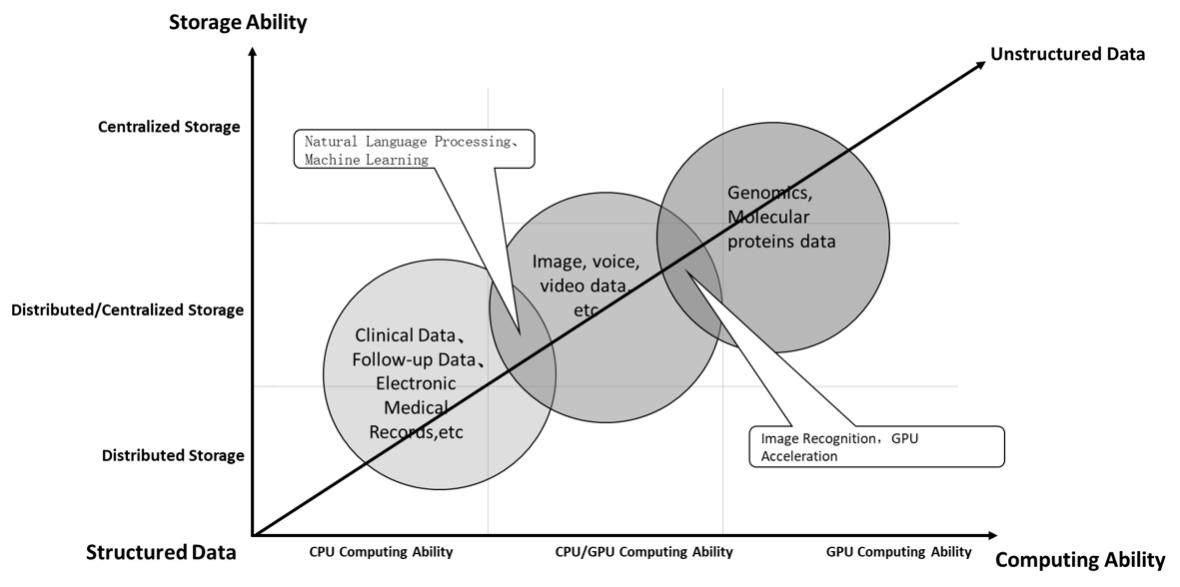
The relationship between the degree of data structuration and storage and computing capability.

Massive data were collectively stored in the storage devices of the WCH-BDP. To better support data analysis, storage and calculation environments were designed and provided according to the characteristics of each data modality. Generally, the amount of data structuration is negatively correlated with storage space and computing power (ie, the greater the data structuration, the lower the requirements for storage space and computing power) (Figure 2). Hence, data that are poorly structured require more storage space and stronger computing power.

Highly structured data do not require much storage space. They can be stored on a distributed storage device, and computations can be effectively run by conventional central processing units.

Semistructured data, such as the records of a patient’s major complaint, disease history, examination findings, and examination conclusions in Chinese electronic medical records do not occupy much storage space and can be stored in distributed storage devices. However, these data require natural language processing for analysis; therefore, both general graphics processing units (GPUs) and central processing units are needed to provide sufficient computing power.

Unstructured data that are large in total volume but small in individual volume (eg, the original image data in DICOM format generated by thin-layer scanning examinations such as radiology and ultrasound) require a relatively high storage space and must be stored in centralized storage devices (mostly network-attached storage) to save resources. To analyze and mine these data, characteristic modeling is performed mainly with machine learning or deep learning technologies, which requires the support of a large amount of GPU power and private GPU resources.

Unstructured data with a large individual volume, such as genomic data, are generally stored directly in object-based storage. Genomic sequencing generates not only a large amount of original sequencing data, but also, a large amount of data related to multiple processes in research on biological information. As a result, genomic data sets often have a tremendous size, and the storage space that is required is mostly measured in petabytes. Analysis and mining of the types of data stored in object-based storage require a lot of clustered computing power supplied by multiple high-performance GPUs, which necessitates the deployment of accelerated supercomputing power supported by GPU clusters.

Traditional big data platforms are planned and implemented in a unified way with storage and computing integration. The design of the WCH-BDP is the realization of storage and computation separation. The WCH-BDP first solves the problem of effective storage and management of massive genomic data files. Second, when researchers use genetic data for analysis, the scheduling software provided by the WCH-BDP loads the analysis data into the supercomputer environment for analysis. After the analysis is completed, the analysis results are retained, and the temporary storage space occupied by the analysis process is released.

### Data Integration

In China, medical institution operations information database systems mainly include Microsoft SQL Server, Oracle database, MySQL database, Caché database, and MongoDB; other unstructured data are mostly stored in the form of files. The WCH-BDP achieves data integration through both real-time and non–real-time data entries. For real-time data integration, a master–slave database is generated, and the log data of the slave database are parsed and captured in real time. Real-time data integration is suitable for an operations information system with mostly structured data. Parsing the information in the slave database does not consume much physical resources and therefore does not affect the performance of the master database, which ensures the security and stability of data integration. Image data integration and entry can be completed by directly reading image files using the DICOM protocol. Genomic data and other data in the form of files are not integrated in real time and are entered via file transfer protocol.

### Master Index Governance

**Figure 3:**
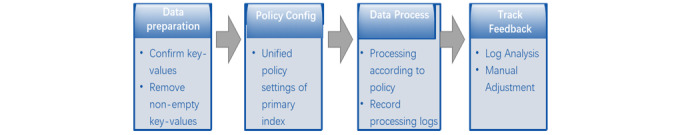
Flowchart of master index governance.

The enterprise master patient index is the unique tag of a patient in a health care institution. One patient might have different enterprise master patient indexes in the different operating systems of a hospital because these systems were constructed independently. This necessitated a master patient index governance to standardize the enterprise master patient indexes. The governance of the master index data is achieved through the master index governance system. The governance system included 4 stages: data preparation, standardization strategy, data processing, and tracking or feedback (Figure 3).

The data governance strategy of the WCH-BDP mainly focused on 3 key values: ID number, name, and telephone number. The configurations and data processing of these 3 key values are shown in the following table (Table 1).

Master index data processing cannot rely only on the system for automatic completion. For example, in the fifth row of Table 1, both the Name and Telephone number are *Equal*, but the ID card number is *Unequal*, which might be because the same patient used a different ID card for registration. In this case, the last step for standardization of the master index is critical, during which the uniqueness of each master index can be ensured by manual adjustment after log analysis or by referring to the actual operational process.

**Table 1:** Governance strategy of enterprise master patient index.

ID number	Name	Telephone number	Result
Equal	Equal	Equal	Accept
Equal	Equal	Unequal	Accept
Equal	Unequal	Equal	Accept
Equal	Unequal	Unequal	Denied
Unequal	Equal	Equal	Accept
Unequal	Equal	Unequal	Denied
Unequal	Unequal	Equal	Denied
Unequal	Unequal	Unequal	Denied

### Master Data Governance

Master data include all related data items listed in the data dictionary, such as health care institution code, drug code, diagnosis code, and anesthesia method [22] (Table 2). Master data governance is the aim to map and handle master data discrepancies caused by different system standards. Classification and reference standards for the master data to be processed are determined, and they are used to map relationships between data in the database. The results are published to users for subscription and application.

**Table 2:** Example of the WCH-BDP master data reference standard.

Classification of master data	Number of reference standards	Example
Classification of diseases	5	ICD-10GB/*t* 14396-2016 Classification and codes of diseasesGB/*t* 15657-1995 Classification and codes of diseases and ZHENG of traditional Chinese medicine
Basic industry information	6	GB 11714-1997 Rules of coding for the representation of organizationGB/*t* 13745-2009 Classification and code of disciplinesGB/*t* 2260-2007 Codes for the administrative divisions of the People's Republic of China
Health informatics	20	GB/*t* 21715-2020 Health informatics—Patient healthcard dataGB/*t* 24465-2009 Health informatics. Health indicators conceptual frameworkGB/*t* 25512-2010 Health informatics-guidelines on data protection to facilitate trans-border flows of personal health informationGB/*t* 30107-2013 Health informatics.HL7 Version 3.Reference information modelGB/Z 24464-2009 Health informatics-Electronic health record-definition, scope and contextGB/Z 28623-2012 Health informatics. Interoperability and compatibility in messaging and communication standards. Key characteristics
Personal information	12	GB/*t* 2261-2003 Classification and codes of basic personal informationGB/*t* 4658-2006 Codes for record of formal schoolingGB/*t* 4761-2008 Codes for family relationshipGB/*t* 6565-2009 Classification and codes of occupationsGB/*t* 8561-2001 Code of professional technical position
Information technology	3	GB/*t* 34960.1-2017 Information technology service－Governance GB/*t* 39725-2020 Information security technology—Guide for health data security

### Metadata Governance

#### Metadata Governance Process

**Figure 4:**
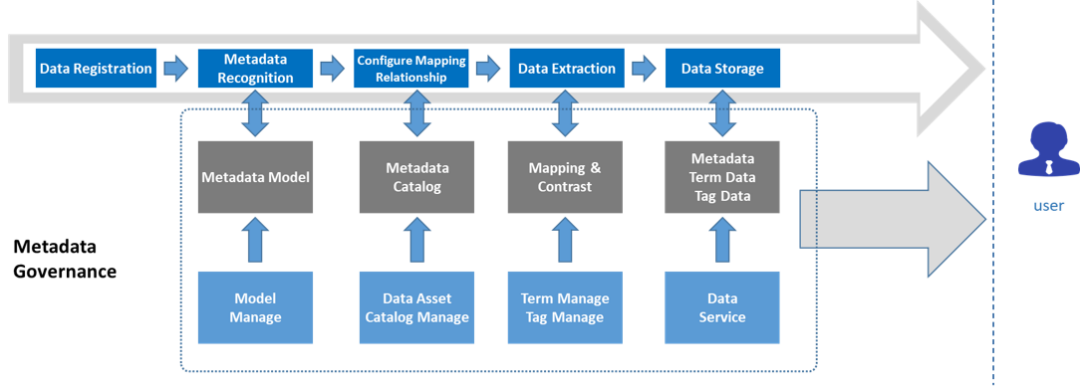
Metadata governance architecture.

Metadata are the bridge between the data and the data users. They describe the content (what), coverage (where, when), quality, management method, owner (who), and provision method (how) of data. The metadata governance approach used by the WCH-BDP is shown in Figure 4.

In the WCH-BDP, a unified metadata model for identifying the registered original data was designed and constructed based on the standards of the Common Warehouse Metamodel of the Object Management Group. Based on the metadata directory, the relationship for data mapping is configured to initiate data extraction. The mapping results are saved in terms of the configured relationship and compared with the standard terminology and tags in the metadata management system. All extracted data are stored in the platform’s data repository, which provides data services to the application layer using standardized metadata, terminology, and tags.

#### Terminology Standardization

Terminology standardization is an important step in metadata governance. The WCH-BDP uses medical terminology from the Chinese Open Medical and Healthcare Alliance to standardize the terminology in the metadata system.

#### Governance of Tag Data

Tagging is also one of the more effective methods used for data governance. Unlike terminology synonyms, the *tag* is an attribute that reflects the application of data [23]. Tag data are more suitable for the preprocessing scenario of comparing or calculating a large amount of data, such as the change in the value of a test result before versus after surgery, or the range of patient blood pressure change before and after taking a medication. The governance of tag data should consider the app objectives and continuously track and maintain tag data.

#### Natural Language Processing Governance

Many medical records and reports with descriptions written in Chinese are difficult to process due to the unique characteristics of the Chinese language. Currently, in China, one mainstream post–structured data processing method is the use of machine learning technology combined with health care terminology to segment words and refine concepts from Chinese text [11]. It is common practice to optimize the word segmentation algorithm model by comparing the word segmentation results with standard terms and aggregating the comparison results, through which a comprehensive corpus and a more accurate word segmentation algorithm can be developed. The algorithm model and the content of the corpus may depend on disease type, regional culture, and even writing habits; therefore, these factors had to be considered during construction.

### Data Security Management

#### Overview

Data utilization and security are contradictory. To ensure proper and secure data use, all countries have issued laws and regulations on the security of health and medical data. Referring to relevant international and domestic laws and regulations on security protection, the WCH-BDP uses a *5S* (ie, data security, app security, use security, management security, and ownership security) control system for data storage, access, use, and transformation of scientific and technological achievements.

#### Data Security

Data security refers to data classification and hierarchical management security. Scholars from Harvard University have proposed that a data classification system should be established to ensure data security according to the laws and regulations of Health Insurance Portability and Accountability Act and related ethical regulations on scientific research [24,25]. In the WCH-BDP, data security has 5 levels, with 3 dimensions: affected object, degree of impact, and scope of impact. The data access interface with corresponding restrictions is provided to each level accordingly (Table 3).

**Table 3:** Data security strategy of the big data platform.

Security classification	Security level	Descriptions	Examples	Precautions
Level 1	Most confidential	Hypersensitive information	Financial data, personal authentication data	A specified environment for a specified single individual to use
Level 2	Confidential	Highly sensitive information	Credit data, personal health privacy data	A specified environment for a specified single role to use
Level 3	Secret	General sensitive information	Personal information during diagnosis and treatment, contract information, employee management data of employees	Use after the role group is authorized
Level 4	Internal use only	Information that is not publicly disclosed	Organizational structure, basic information of employees, general data after desensitization	Use after internal authorization
Level 5	Open to the public	Data that can be publicly disclosed	Summarized results obtained by statistical analysis	Public access or use

#### App Security

App security refers to the data app security management of a system during its processes. In the WCH-BDP, a unified master data authentication service is provided for user authentication so that each app system manages only role and app permissions required by the system itself. When an actual role user uses an app, data desensitization and encryption are completed through the unified platform service interface to ensure the security of the data app.

#### Use Security

Use security refers to the security management during the processes of data processing, use, and analysis. The National Institutes of Health has emphasized that special attention should be paid to privacy protection in data apps [26]. Patient information that needs to be desensitized generally includes all of the information that can be directly and indirectly linked to or used to locate the patient, including name, ID, telephone number, address, contact information, information related to infectious disease [27]. The WCH-BDP ensures patient privacy protection through desensitization, encryption (Table 4), and multiparty computation.

Multiparty computation is a controllable and measurable method proposed by a Turing Award winner, Qizhi Yao, to solve the problem of data abuse [28]. Before statistical analysis of massive data, the multiparty computation service gateways in the WCH-BDP are deployed to complete all data calculations in the security domain and provide the final calculation results to users through the platform service. This process can effectively manage data use security.

**Table 4:** Data desensitization and encryption policies of big data platform.

Name of strategy	Scope of data involved	Design of desensitization and encryption policy
Digital data	Operating revenue, key quantity...	Fuzzy rounding method or fuzzy percentage method
Structured data of fixed length	Identification card number, telephone number, name...	Replace or encrypt from the starting bit to the end of the specified length range
Text data of varying length	Address, electronic medical record, descriptive diagnosis (infectious disease)	Locating sensitive content, then replace or encrypt sensitive characters
Image data	Radiological, ultrasonic and pathological imaging data	In image files, encryption algorithm is used for desensitization and watermarking is configured
File data	Genomics, molecular protein data	Locating sensitive content and rename sensitive characters

#### Management Security

Management security of platform data requires that all platform services, including data services, be completed under secure closed-loop management. In the WCH-BDP, all tasks—data access, management, and governance; data analysis, utilization, and mining; and the transformation and realization of scientific research findings—are completed through platform services for data resources, storage, computing power, and network access. Establishment of the management security system can effectively guarantee the security of the data utilization process.

#### Ownership Security

Currently, most research findings are obtained in a laboratory or scientific research setting but are difficult to apply in an actual production setting, which directly leads to a low transformation rate of research achievements and insufficient recognition of intellectual property rights. In the WCH-BDP, through the integrated service gateway that connects to the real service operation scenario, published research findings can be directly introduced into clinical practice, realizing the translation of scientific research to the clinic. In this process, the design of the integrated service gateway management system can effectively ensure researcher’s ownership security.

### Data Quality Management

#### Overview

Data quality management is a continuous process and an important link in data governance. Health care data quality management in the WCH-BDP involves the following steps: (1) building a data quality standard system; (2) evaluating current data quality; (3) analyzing data quality problems; (4) developing and optimizing the solutions in detail to solve problems; and (5) establishing a data quality control knowledge database for future reference. The steps above are continuously iterated to form a closed-loop system for health care data quality management.

#### Standards of Data Quality

Common data quality problems include data outliers, duplications, data without any clear relationships, orphaned data records, and data that make no logical sense medically. Fully referring to the evaluation and grading standards of electronic medical records issued by the National Health Commission of China and research findings by experts in the field of health care [29-31], we developed a standard system for data quality control using the dimensions *consistency*, *integrity*, *integrability*, *timeliness*, and *stability* as parameters for evaluation (Table 5).

**Table 5:** Data quality standard system.

Dimension	Content of the dimension	Quality indicator	Rule
Consistency	Check whether the data value is in the dictionary domain	Consistency rate	Higher than 90%
Integrality	Check the completeness of the required data	Percentage of integrality	Higher than 80%
Relevance	Check the relationship between key data	Degree of relevance	Higher than 95%
Timeliness	Check the logical validity of time-type data	Timeliness ratio	Higher than 80%
Uniqueness	Check whether data duplication exists	Repetitive rate	Lower than 0.01%
Stability	Check whether the fluctuation of data volume is abnormal	Fluctuation ratio	Lower than 20%

#### Process Quality Control

In addition to data quality control for original data, quality control management of procedures in operations are included [21]. Data quality problems reflect problems in operations processes. By formulating a plan that optimized data quality by improving management and operational protocols, the WCH-BDP achieved its goal of controlling the quality of operations to obtain high-quality data outputs. Data quality management is a continuous optimization process that requires the participation of all personnel involved.

### Data Service Support

The WCH-BDP is an environment that can effectively support data services with voluminous data repositories and strong physical resources for data computation and storage. Under an overall ecology of data security management, the platform can provide support to data services such as computing, storage, and virtualization. Search engine services, terminology services, and artificial intelligence services can be effectively integrated to meet the needs of all health care operations.

## Results

### General

The WCH-BDP was formally launched in November 2020, after 3 years of construction, for demand surveys, platform design, module development, and pilot operation. To date, the platform has formed a massive high-dimensional database with more than 12.49 million patients, 75.67 million visits, and 8475 data variables. The platform has brought hospital informatization to a new level and greatly improves the hospital’s overall big data capabilities.

### Computing Power

The WCH-BDP has more than 20 PB data storage capacity and runs on a server cluster consisting of 252 physical servers: 117 servers are dedicated to computing and analyzing, and 38 are equipped with more than one GPU card. In the server cluster dedicated to computing, there are 320 CPU cores and 149 GPU cards. In total, the platform has computing powers that exceed 300 TFLOPS for clinic data, 600 TFLOPS for image data, and 1000 TFLOPS for genomic data.

### Scope of Data Integration

The WCH-BDP integrates diagnosis and treatment data from clinical information systems, clinical research data from science and research information systems, and management activity data from management information systems. Of 134 hospital systems, the platform has integrated the data of 27 systems, including hospital information systems, laboratory information systems, radiology, ultrasound, web-based medical appointments, human resources, equipment and supply management, Internet medical service since January 2008. In addition, all image data in the picture and communication systems and more than 20,000 patients’ genomic data have been entered into the platform.

### Database

Using Fast Healthcare Interoperability Resources Reference Information Model 3.0 standards, data integrated into the WCH-BDP were reorganized and arranged based on the type of operational activities while considering the characteristics of China’s medical system. Finally, a database that includes 134 data charts for 18 subject fields was developed. The top 5 subject fields that were responsible for the most data in the database are listed in Table 6. To date, the database includes 8475 data variables and 6.272 billion rows of data records.

**Table 6:** Top-5 subject fields in terms of number of data rows.

Number	Subject field	Tables, n	Data variables, n	Rows (10,000×n)	Systems involved
1	Medical record	10	476	369824.53	Hospital information system, online diagnosis and treatment system
2	Medical technology	6	502	115073.35	Electrocardiogram system, radiology information system, echocardiography system, endoscopic system, dynamic electrocardiogram system, pathology information system, ultrasonic system, laboratory information system, interventional surgical workstation, medical technology reservation information system, outpatient information system, physical examination information system
3	Fee	8	440	60855.24	Hospital information system, physical examination information system, online diagnosis and treatment system
4	Medical advice	3	395	21366.64	Hospital information system, online diagnosis and treatment system
5	Staff	13	802	18138.94	Hospital information system, physical examination information system, electronic data capture, human resource system

### Data Asset Directory

The platform has constructed a data asset directory for users by further standardizing the data through the governance of master data, metadata, terminology, and natural language processing. To ease the process of inquiry, the directory has a hierarchical tree structure that allows users to select the data items that they need using fuzzy searches. To minimize comprehension difficulties, the directory uses a data variable–naming scheme consistent with the names in the interface of the corresponding operations systems as much as possible; in addition, users can see the information of the data source, content, and range of values.

The data asset directory covers 13 fields and 1488 data variables (Table 7). Of the 13 fields, the original data variables in 9 fields are structured variables, and those in 3 fields (medical records, imaging examinations, and nursing care records) are semistructured text. Semistructured data were converted into poststructured derived variables by using approaches such as word segmentation, entity extraction, and semantic identification. For patient tag information, the app-oriented poststructured derived variables were generated using a data-mining algorithm.

**Table 7:** List of data assets.

Number	Directory field	Data variable (unit)	Types of variables	Example
1	Demographic	91	Structured	Gender, age, occupation, present address, nationality, height, weight, blood type
2	Basic medical information	410	Structured	Appointment date, visit date, clinic department, clinic type, supervising doctor, the department transferred to, admission date, discharge date, discharge state
3	Medical record information	123	Unstructured	Admit note, progress note, discharge record
4	Clinical diagnostic information	47	Structured	Clinic diagnosis, emergency diagnosis, admitting diagnosis, discharge diagnosis, medical insurance diagnosis, pathological diagnosis
5	Surgical and operational information	138	Structured	Name of operation, name of procedure, surgeon, surgical grade, anesthesia grading, incision type, level of healing
6	Diagnosis and treatment information	166	Structured	Type of medical order, drug name, usage, dosage, frequency, execution time of medical order
7	Laboratory test data	147	Structured	White blood cell count, red blood cell count, sodium level, uric acid level, blood glucose level, creatinine level
8	Imaging results	124	Unstructured	Magnetic resonance imaging, computed tomography, x-ray, ultrasound, digital radiography
9	Nursing record information	53	Unstructured	Admission assessment, daily records, nursing records
10	Physiological monitoring data	50	Structured	Vital signs
11	Scale evaluation data	50	Structured	Mood index, pressure ulcer assessment, risk assessment of falling out of bed
12	Medical cost information	65	Structured	Name of charge items, amount of charge items, settlement time
13	Patient label information	24	Unstructured	Patients with lower test results after surgery, patients with higher blood pressure after medication

### Assessment of WCH-BDP Performance

#### Service Support to Major Projects

Supported by the data services of the platform, the research teams of WCH have constructed more than 120 disease databases for different research aims, including a number of national-level multicenter disease databases. Since it was launched, the platform has supported more than 20 clinical and hospital management research projects, of which, 3 have won second prize in the State Science and Technology Progress Award of China and first and second prizes in the Sichuan Provincial Science and Technology Progress Award.

The platform’s support of basic research is exemplified by a project on the mechanisms of allosteric regulation and signal transduction of G protein–coupled receptors conducted by the State Key Laboratory of Sichuan University; using the storage and computing power provided by the WCH-BDP, the research team revealed the microconversions of key amino acids during allosteric regulation, which laid the foundation for the design and screening of G protein–coupled receptor–targeting small molecule allosteric regulators (unpublished, X. Yang, PhD, 2022).

Another example of the platform’s support of clinical research is a lung cancer research project at WCH. Using the data resources, storage resources, computing power, and exploration environment provided by the platform, the lung cancer research team identified and validated high-sensitivity high-specificity markers for early diagnosis of lung cancer [32], and the team further developed the first lung cancer database and artificial intelligence–assisted product for lung nodule diagnosis in China [33]; these papers [32,33] are listed in Essential Science Indicators—published in *Cell* (impact factor 41.582) and *Signal Transduction and Targeted Therapy* (impact factor 18.187). Research results have also been published in internationally renowned academic journals, such as *Medical Image Analysis* (impact factor 11.148) [34] and *Nature Biomedical Engineering* (impact factor 25.671) [35].

Artificial intelligence–assisted products for lung nodule diagnosis can detect 3 mm to 5 mm pulmonary nodules with 98.8% accuracy through artificial intelligence technology, which was significantly better than the performance of domestic and foreign specialists (79.9% of doctors with senior experience in Peking Union Medical College, only 40.9% of those with junior experience), and the average reading of each chest computed tomography (CT) can save 3 to 5 minutes [33]. In 2020, the system was used in more than 100 hospitals nationwide, including in West China Hospital of Sichuan University. It not only improves the efficiency of chest CT image reading, but also reduces the rate of missed diagnosis of small pulmonary nodules. It also plays an important role in realizing the homogeneity of early diagnosis of lung cancer.

Using this platform, research on segmentation of adrenal glands from CT images [36], in which a novel 2-stage deep neural network for adrenal gland segmentation in an end-to-end fashion was proposed, used data resources, storage resources, computing capabilities, and the exploration environment provided by the WCH-BDP platform. The research data set contained 348 CT volumes acquired from 348 patients, which was used to verify the performance of the new method and show that the new cascaded framework outperformed, with respect to accuracy, state-of-the-art deep learning in segmenting the adrenal gland [36].

#### Changes in the Conventional Data Service Model

The launch of the WCH-BDP has led to tremendous changes in the data service model of the hospital (Table 8). Users used to rely on information systems personnel for data use, but now they can analyze data by themselves throughout the entire process. Using the search engine service provided by the platform, researchers can quickly retrieve data from the databases to form a disease database suitable for real-world research, which is then introduced into the information exploration environment for data statistical analysis and mining.

**Table 8:** Changes between the traditional and the present data service.

	Traditional data services	Data services base on the platform
Data visualization	Data not visible	Users can visually view the available data catalog
Data retrieval	Data engineers develop code through experience	Users can customize the search format and output format through the search engine and preview the results
Data approval	Data are available after ethical review and clinical study program approval	Data are available after ethical review and clinical study program approval
Data mining	Use your own computer to analyze data	Development environment and tools, such as R, SPSS, and Python, can be used on the platform, and computing power provided by the platform can be called by data mining algorithm
Data download and access	Download the data which data engineers develop and perform encryption	The platform creates accounts with different permissions for registered users. In a network environment after security authentication, authorized users can log in to the big data platform unified portal through virtual desktop infrastructure. The platform provides each authorized user with private storage space of different capacities. Users can directly store their research results in this space, or install the software developed by our college on their personal computers to transfer the research results to personal computers

The launch of the WCH-BDP has substantially improved the capacity of data services (Table 9). The scope of usable data in the platform is 3.37 times that of the previous level, an increase from 8 operation systems covered by the previous data warehouse to 27 systems by the big data platform. The dimensions of usable data are 1.8 times those of the previous level, an increase from 803 data variables to 1488 variables. The amount of usable data is 2.4 times the previous level, increasing from 6.8 billion rows of data records to 16.49 billion rows.

The engineers completed 996 instances of manual data service in the 6 months before the launch of the platform (monthly mean 166). In contrast, 8561 self-service data retrievals were completed each month after the launch of the platform—a 51-fold increase in service efficiency. Each person could complete 2 instances of data services each day before but could complete 65 instances per day after the launch of the platform due to the help offered by the automated search engine—a 37-fold increase. In addition, the platform shortened the average duration of each data service 30-fold, from 4.5 hours to 0.15 hours. The platform substantially improved the volume and efficiency of data services.

**Table 9:** Comparison of data service capabilities.

	Before the launch	After the launch
Number of business systems covered	8	27
Data dimension	803	1488
Data volume (billion)	6.8	16.49
Number of monthly services	166	8561
Time per request (hour)	4.5	0.15

#### Improvement of Data App Security

Data security is a key focus of the platform. In the past, data security relied mostly on the professional ethics of data engineers. In contrast, in this platform, which automatically manages data and provides data services, data security management is mostly system-based and manually assisted because all operation activities leave footprints in the system. This can prevent the abuse of individual permissions and effectively ensure data security.

## Discussion

The performance of the WCH-BDP has demonstrated that data resources can be effectively converged and governed to form highly usable data assets that have extremely high application value in the field.

The success of the construction of a big data platform in health care is based on the following: (1) The project is managed with a strong organizational structure that has a top-down data governance committee involving multiple parties. The committee leads and oversees data governance duties. (2) The project is led by an information technology department that can provide technical support. The information technology team should have excellent skills and be familiar with the operations and data connotations of all systems in the hospital. (3) All departments and sections of the hospital should participate in the project, and detailed demand surveys and analyses should be performed. (4) The project requires sufficient scientific knowledge in medical informatics, management, and engineering to ensure smooth integration of medicine, management, and informatics for overall framework design. (5) The ethics office and clinical research management sections of the hospital should participate in the project to ensure patient privacy protection and data security. (6) The construction project needs suppliers who have rich experience and can provide sufficient technical support. (7) A sufficient amount of servers are needed for data storage and computation. (8) The project needs sufficient financial support.

Similarities between the WCH-BDP and other data platforms [1-14] are that they provide data services through data integration, need to complete data integration, have various medical data, can provide structured data services, and can provide massive data retrieval. However, there are 6 differences: (1) The WCH-BDP integrates all business system data, while most other platforms integrate data on demand. (2) Our platform parses database logs and migrates full business data to the data center in a master–slave database synchronization mode. Some of the platforms use API interfaces to implement data migration. (3) The WCH-BDP accesses data constantly and in real time. Nevertheless, some other data platforms often access data by the day. (4) While most other data platforms only integrate clinical data, the WCH-BDP integrates both clinical data and hospital management data. (5) Most other data platform may not have supercomputing capability. After integrating supercomputing capability, the WCH-BDP can store more than 20 PB and calculate faster than 1900 TFLOPS. (6) Most other platforms can only provide conventional data storage and processing functions by disease type. The WCH-BDP provides an analysis environment equipped with data mining tools, including open-source tools, such as R and Python, and paid apps, such as SAS and SPSS. Researchers can use distributed clusters for data mining.

The WCH-BDP can be further improved and optimized by (1) connecting more operations systems of the hospital to the platform and continuously optimizing the data governance strategies; (2) further utilizing and mining the data (eg, exploring multimodal artificial intelligence apps in health care); (3) making the platform a multicenter public platform by launching transdisciplinary, cross-hospital, cross-regional collaborations and including more medical information data; and (4) providing the hospital with further full-cycle *standardization + security + service* big data services.

## Abbreviations

CT: computed tomography
GPU: graphics processing unit
WCH: West China Hospital of Sichuan University
WCH-BDP: West China Hospital of Sichuan University big data platform
